# The clinicopathological PANOMEN-3 classification predicts pituitary adenoma prognosis: a real-world retrospective single center study of a surgically treated cohort

**DOI:** 10.1007/s11102-025-01562-9

**Published:** 2025-09-07

**Authors:** Sabrina Chiloiro, Antonella Giampietro, Raffaele Migliore, Chiara Palumbo, Penelope Giambò, Flavia Costanza, Pier Paolo Mattogno, Rosalinda Calandrelli, Tommaso Tartaglione, Liverana Lauretti, Mario Rigante, Marco Gessi, Simona Gaudino, Laura De Marinis, Antonio Bianchi, Francesco Doglietto, Alfredo Pontecorvi

**Affiliations:** 1 Facoltà Di Medicina E Chirurgia, Università Cattolica del Sacro Cuore, Rome, Italy; 2 Dipartimento Di Medicina Interna, Endocrinologia E Diabetologia, Fondazione Policlinico Universitario A. Gemelli, Istituto Di Ricovero E Cura a Carattere Scientifico (IRCCS), Rome, Italy; 3 Dipartimento Di Neurochirurgia, Fondazione Policlinico Universitario A. Gemelli, Istituto Di Ricovero E Cura a Carattere Scientifico (IRCCS), Rome, Italy; 4 Dipartimento Di Scienze Radiologiche, Fondazione Policlinico Universitario A. Gemelli, Istituto Di Ricovero E Cura a Carattere Scientifico (IRCCS), Rome, Italy; 5 Dipartimento di Neuroscienze, Organi di Senso e Torace, Fondazione Policlinico Universitario A. Gemelli, Istituto Di Ricovero E Cura a Carattere Scientifico (IRCCS), Rome, Italy; 6 Unità di Neuropatologia, Fondazione Policlinico Universitario A. Gemelli, Istituto Di Ricovero E Cura a Carattere Scientifico (IRCCS), Rome, Italy

**Keywords:** Acromegaly, PitNET, Prolactinoma, Cushing, Neuroendocrine tumors

## Abstract

**Introduction:**

Pituitary adenomas (PAs) are generally benign neoplasms, though in rare cases may exhibit aggressive behavior. In 2024, the PANOMEN-3 workshop released a new clinical-pathological classification. The objective of this study was to examine the potential of the PANOMEN-3 classification to predict prognosis of PAs and guide treatment in our single center cohort of patients with PAs.

**Patients and methods:**

A longitudinal, retrospective, observational study was performed on patients with a PA diagnosis. The PANOMEN 3 classification was applied to each patient 6 months after surgery. Resultant grades were correlated with surgical outcome, disease recurrence or progression.

**Results:**

289 patients were included. According to the PANOMEN-3 classification, 9 patients (3.1%) were designated as grade 0, 101 patients as grade 1 (34.9%), 140 patients as grade 2 (48.4%) and 39 patients as a grade 3 (13.5%). At last follow-up assessment, 186 patients were found to be disease-free (64.4%), 93 patients (32.5%) exhibited a stable residual, 9 patients (3.1%) had recurrence and/or progression of their PA. The risk of recurrent/residual disease was increased in grade 1 (OR: OR:1.4 95%IC: 1.2–1.7), grade 2 (OR:1.5 95%IC: 1.2–1.9) and grade 3 (OR:5.7 95%IC: 2.7–12.5). Grades 1, 2 and 3 were associated with a shorter disease-free survival interval as compared to those with a grade 0 PANOMEN-3 score.

**Conclusion:**

The PANOMEN-3 score is useful in clinical practice, aiding physicians to better plan patient follow-up, as well as to manage residual disease and treatment strategies post-surgery.

## Introduction

Pituitary adenomas (PAs) arising from cells of the anterior pituitary gland and are associated with both local and peripheral clinical sequelae [1]. Although almost invariably benign, they may sometimes exhibit invasive features [2, 3]. Among clinically significant pituitary adenomas, about 30 to 50% of cases show invasion of adjacent structures, nearly 20% show recurrence or progression after initial surgery and < 2% exhibit aggressive behavior, defined as resistance to conventional therapy and/or a rapid regrowth [2, 3]. The prevalence of PAs among the general population is estimated to 115 cases per 100.000 inhabitants [1]. Early detection of aggressive PAs plays a crucial role in identifying patients at high risk for disease recurrence/progression and those likely to be poorly responsive to standard therapies. In 2018, the European Society of Endocrinology established a definition for aggressive PAs as those exhibiting atypically rapid and invasive growth, resistance to standard medical therapies, development of recurrences after surgery and radiotherapy, and specific histopathological features, including the presence of mitoses, a high proliferative index, and the expression of p53 [3, 4].

The prevalence of aggressive PAs remains uncertain [3], and the prognosis of PAs remains a subject of both scientific and clinical interest.

In 2013, a five-tiered clinic-pathological classification was proposed based on evaluation of invasiveness and proliferation [5]. In 2017, the WHO proposed an integration of the nomenclature of neuroendocrine tumor into the standard PA classification, with the objective of achieving a more precise description of the biological, molecular, and clinical heterogeneity of PAs [6]. In 2022 the WHO proposed a novel histopathological classification of PAs, thereby identifying and defining more aggressive histotypes, including Crooke cell, silent corticotroph, sparsely granulated somatotroph, null cell, acidophil stem cell and immature PIT1-lineage adenomas [7, 8]. However, the prognostic classification of PAs proposed by WHO is limited to those PAs undergoing surgical resection, accounting for less than half of such lesions. Furthermore, the WHO classification does not consider clinical features, such as invasive growth, the extent of surgical excision, and the presence of genetic syndromes that may affect the overall disease outcome. A comprehensive clinical classification integrating clinical, genetic, biochemical, radiological, pathological and molecular information remained required to guide prognosis, therapy and outcomes for all patients with PAs [9, 10]. In 2024, the Pituitary Society sponsored the pituitary adenoma nomenclature 3 clinical workshop (PANOMEN-3), that proposed an integrated clinical and pathological classification for pituitary neoplasms, with the purpose to predict PA prognosis and outcome [11]. To date, the PANOMEN 3 classification has not yet been validated through prospective cohort studies [12]. Recently a multicenter, Spanish national cohort study demonstrated that increased PANOMEN-3 grades correlated with the recurrence/progression rates [10].

The objective of this study is to examine the efficacy of the PANOMEN 3 classification system in predicting PA outcomes and in stratifying the risk of PA recurrence or progression.

### Patients and methods

A longitudinal, retrospective, observational and single center study was conducted on patients diagnosed with pituitary adenoma diagnosed and subsequently followed at the Pituitary Unit and at the Department of Neurosurgery at Gemelli University Hospital.

### Objectives

The primary objective of the study was to assess the utility of the PANOMEN-3 classification in predicting the outcome of pituitary adenomas, classified as recurrent disease, residual disease, or disease-free.

As a secondary objective, we investigated additional risk determinants of outcome for pituitary adenoma. This included gender, age at diagnosis, histopathology classification according to the 2022 5th edition of the WHO Classification of Endocrine and Neuroendocrine Tumors, clinical assessment, proliferative index (Mib-1), p53 expression, number of mitoses, and number of surgical resections.

### Inclusion/exclusion criteria

Patients harboring a pituitary adenoma were consecutively enrolled in the study, according to the following inclusion criteria:

1. Ascertained diagnosis of pituitary adenoma, with pathology diagnosis in accordance with the 2022 5th edition of the WHO Classification of Endocrine and Neuroendocrine Tumours;
2. Age > 18 years;
3. Neurosurgical resection performed for removal of pituitary adenoma at the Neurosurgery Division at Gemelli University hospital;
4. Data availability related to the study;
5. At least 12 consecutive months of follow-up.

Patients with seller apoplexy were excluded from the study.

### Data collection

Data was collected from patient medical records including gender, age, histology and clinical tumor subtype, proliferative index (Mib-1), p53 expression, number of mitoses, history of previous surgical resection, history of pre-surgical medical therapy, diabetes mellitus, arterial hypertension, major cardiovascular events, second neoplasia, osteoporosis, post-surgical treatments according to tumor subtypes (dopamine agonist [DA], somatostatin receptor ligand [SRL], growth hormone receptor antagonist [GHRa] and steroidogenesis inhibitors), PANOMEN-3 grades, disease outcome at last follow-up and disease-free survival.

Histology tumor subtypes subtypes were defined according to the 2022 5th edition of the WHO Classification of Endocrine and Neuroendocrine Tumours. Clinical tumor subtypes were classified according to scientific society guidelines [13–17].

The PANOMEN-3 grades were determined 6 months after surgery, by assigning an established score to each factor as published [11]:

- phenotype: score 0 for non-functioning pituitary adenoma and prolactinoma, score 1 for acromegaly, thyrotropin-secreting pituitary adenoma, score 2 for Cushing’s disease;
- secretory status: score 0 for normal or biochemically controlled, and score 1 for not biochemically controlled (elevated);
- hypopituitarism: score 0 for absent, score 1 for partial with no adrenal or arginine vasopressin deficiency, and score 2 for hypopituitarism with adrenal or arginine vasopressin deficiency;
- size: score 0 for microadenoma (maximum diameter < 10 mm), score 1 for macroadenoma (maximum diameter ranging from 10 to 39 mm), score 2 for giant adenoma (maximum diameter > 40 mm);
- mass effect: score 0 for absence of symptoms due to mass effect, score 1 for presence of visual field defect and/or cranial nerve palsy; score 3 for cerebrospinal fluid (CSF) leak;
- invasion: score 0 for not-invasive tumors, score 1 for tumors with invasion of the cavernous sinus (Knosp grade 3 and 4);
- residual tumor: score 0 for absence of residual tumor and score 1 for presence of residual tumor;
- histopathology: score 1 for high-risk subtypes (such as immature PIT1-lineage, Crooke cell, null cell, sparsely granulated somatotroph, and acidophilic stem-cell adenomas), and score 1 for tumors with increased proliferation (such as those with at least two mitoses per ten high power and Ki67 > 10%)
- genetic syndrome: score 0 for absent and score 1 for present genetic syndrome, due to germline mutation.

The sum of the scores attributed to all evaluated factors was divided by the number of risk factors assessed to derive a corrected score. Corrected scores were divided into the established grades (grade 0: corrected score 0; grade 1: corrected score higher than 0 and lower than 0.3; grade 2: corrected score equal/higher than 0.3 and equal/lower than 0.6; grade 3: corrected higher than 0.6).

Disease outcome was classified at last follow-up as disease free/cure, residual disease, or recurrent disease, according to last available expert consensus and guidelines [13–19]. Disease-free was defined in patients with radical adenoma excision and normalization of hormone levels in secreting pituitary adenomas, and no disease recurrence at the time of the last follow-up. Residual disease was defined in patients who underwent partial excision of the pituitary adenoma. This definition was based on the presence of a tumor remnant at surgery and/or at MRI imaging performed 3 months after surgery.

Recurrent disease was defined, in functioning pituitary adenomas, as hormonal and/or radiological disease reactivation in patients who had undergone radical adenoma excision and with at least a 6-month disease-free survival. More in detail, in patients with acromegaly, recurrence was defined in cases with new-onset symptoms of acromegaly and increased levels of IGF-I (upper the age-corrected limits of normality) with/without reappearance of recurrent pituitary adenoma. In patients with TSH-secreting pituitary adenoma, recurrence was defined for new-onset symptoms and signs of hyperthyroidism and biochemical confirmation of central hyperthyroidism, with/without reappearance of recurrent pituitary adenoma. In patients with Cushing’s disease, recurrence was defined for new-onset symptoms and signs of hyperthyroidism and biochemical confirmation of ACTH-dependent hypercortisolism, with/without reappearance of recurrent pituitary adenoma. In patients with prolactinoma, recurrence was defined for new-onset symptoms and signs of hyperprolactinemia in association with elevated levels of PRL, with/without reappearance of recurrent pituitary adenoma [13–19]. In all patients with suspected recurrence of hyperprolactinemia, other cases of not-neoplastic hyperprolactinemia were excluded, such as condition of physiologic hypersecretion (pregnancy, lactation, chest wall stimulation, sleep, stress), hypothalamic-pituitary stalk damage (empty sella, pituitary stalk effect, irradiation), systemic disease (chronic renal failure, hypothyroidism, cirrhosis, epileptic seizures) and drugs (dopamine receptor blockers, dopamine synthesis inhibitors, catecholamine depletors, opiates, serotonin reuptake inhibitors, and calcium channel blockers) [20]. In non-functioning pituitary adenomas (NFPAs), recurrence was defined as radiological evidence of disease reactivation during follow-up in patients who had undergone adenoma excision and with at least a 6-month disease-free survival. Disease free survival referred to the time from surgery until the recurrence of the disease, in patients with recurrent disease, and from the surgical procedure to the most recent follow-up in patients with complete recovery. Medical treatment for PAs was established based on the most recent available guidelines and expert position statements [13–19].

### Statistical analysis

Descriptive statistics were used to describe the clinical and demographic characteristics of the patient cohort. The normality of continuous variables was tested using the Kolmogorov–Smirnov test. Quantitative variables were expressed as median and interquartile range and qualitative variables as absolute and percentage frequencies. Chi-squared test and Mann–Whitney non-parametric tests were used to compare categorical and quantitative unpaired data. The independent risk factors for disease outcome were identified from univariable and multivariable logistic regression analyses. All variables were subjected to univariable logistic analysis, and covariates found to be associated (exploratory univariate P < 0.05) with rejection were entered into a multivariable logistic regression model. A stepwise selection method (P < 0.05) was applied to identify the final regression model. Kaplan–Meier survival analysis was performed to investigate the ability of the PANOMEN-3 score in predicting disease-free survival. Analyses were performed with SPSS software version 24.0 for Windows.

## Results

**Fig. 1:**
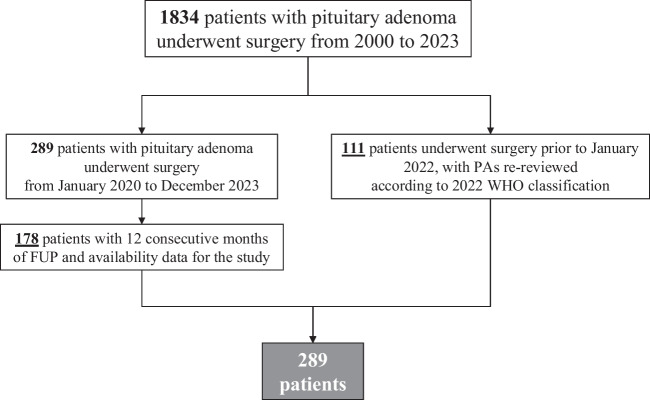
Flow-chart summarizing the study population criteria

A total of 289 patients were included in the study. One hundred seventy-eight patients underwent surgery between January 2022 and December 2023; instead, 111 patients underwent surgery prior to January 2022, and pituitary adenoma samples were re-reviewed by the pathologist for clinical reasons and re-classified according to the 2022 5th edition of the WHO Classification of Endocrine and Neuroendocrine Tumours. Figure 1 summarized the selection of the study cohort. 148 were males (51.2%). The median age at diagnosis was 54 years (IQR: 20). 153 patients had a non-secreting adenoma (52.9%), 65 patients (22.5%) had acromegaly, 34 patients (11.8%) had Cushing’s Disease, 34 patients had a prolactin secreting adenoma (11.8%) and 3 patients (1%) a TSH-secreting adenoma.

Fifty patients had a microadenoma (17.3%), 227 patients had a macroadenoma (78.5%) and 12 patients harbored a giant adenoma (4.2%). Seventy-eight adenomas (27%) invaded the cavernous sinus (Knosp 3 or 4 grades).

PANOMEN-3 classification was applied six months after surgery for those included in the study. Secretory status was classified as normal or biochemically controlled in 241 patients (83.4%), and biochemically active in forty-eight patients (16.6%). 28 patients with biochemical active disease had acromegaly (58.3%), 5 had Cushing disease (10.4%) and 15 had prolactin secreting adenomas (31.3%). 24 patients had partial hypopituitarism (excluding adrenal and arginine-vasopressin deficits), and 102 patients (35.8%) had hypopituitarism (including adrenal and arginine-vasopressin deficits). The remaining 163 patients (56.4%) had normal pituitary function. Surgery was considered radical in 183 patients (63.3%), while 106 patients had tissue residual after surgery (36.7%).

One hundred twenty-three pathological specimens (42.6%) were classified as gonadotroph adenomas, 58 (20%) as somatotroph adenomas, 53 (18.3%) as corticotroph adenomas, 34 (11.8%) as lactotroph adenomas, 7 (2.4%) as thyrotroph adenomas, 2 (0.7%) as mammosomatotroph adenomas and one (0.3%) as an immature Pit-1 lineage tumor. According to the PANOMEN-3 classification, 24 adenomas (8.3%) were identified as high-risk and high proliferative subtypes. Ten lesions were diagnosed as sparsely granulated somatotroph adenomas (3.5%), one as Crooke cell (0.3%), one as immature Pit1-lineage tumor (0.3%), one (0.3%) had a proliferative index (Mib-1) > 10% and eleven (3.8%) had at least two mitoses per ten high power fields. A genetic syndrome was diagnosed in two patients: a patient was diagnosed with MEN1 and a patient with FIPA syndrome.

According to the PANOMEN-3 classification, 9 patients (3.1%) had a grade 0, 101 patients had a grade 1 (34.9%), 140 patients had a grade 2 (48.4%) and 39 patients had a grade 3 (13.5%).

At last follow-up, pituitary adenoma recurrence/progression occurred in 9 patients (3.1%), and death related to the disease occurred in 3 of these 9 patients. At last follow-up, 186 patients were found to be disease-free (64.4%), and 94 patients (32.5%) had stable residual disease. In the entire study cohort, 237 patients (82%) were in remission after surgery and remained on active observation, 23 patients (8%) were on treatment with dopamine agonist, 15 patients with first generation somatostatin receptor ligand (5.2%), 7 patients (2.4%) with second generation somatostatin receptor ligand (Pasireotide Lar) or GHR antagonist (pegvisomant monotherapy), 4 patients (1.4%) with steroidogenesis inhibitors and 6 patients (2.1%) with combination treatments. Median duration of follow-up was 34 months (ranges: 12–84).

Forty-seven patients had mellitus diabetes (16.3%), 108 arterial hypertension (37.4%), 36 osteoporosis (12.5%), 26 were diagnosed with an additional neoplasm (9%) and 8 patients had a major cardiovascular event (2.8%).

The PANOMEN-3 classification did not predict occurrence of diabetes mellitus (*p* = 0.114), arterial hypertension (*p* = 0.013), major cardiovascular events (*p* = 0.126), osteoporosis (*p* = 0.261) and second neoplasm (*p* = 0.07), as shown in Table 1.

**Table 1:** Frequency of comorbidities according to PANOMEN-3 grades. Univariate analysis

	Grade 0	Grade 1	Grade 2	Grade 3	*p*-value
Diabetes Mellitus					0.114
No n, (%) Yes n, (%)	9 (3.7%) 0 (0%)	89 (36.8%) 12 (25.5%)	115 (47.5%) 25 (53.2%)	29 (12%) 10 (21.3%)	
Arterial hypertension					0.013
No n, (%) Yes n, (%)	8 (4.4%) 1 (0.9%)	73 (40.3%) 28 (25.9%)	76 (42%) 64 (59.3%)	24 (13.3%) 15 (13.9%)	
Major cardiovascular events					0.126
No n, (%) Yes n, (%)	9 (3.2%) 0 (0%)	101 (35.9%) 0 (0%)	133 (47.3%) 7 (87.5%)	38 (13.5%) 1 (12.5%)	
Second neoplasm					0.07
No n, (%) Yes n, (%)	9 (3.4%) 0 (0%)	87 (33.1%) 14 (53.8%)	133 (50.6%) 7 (26.9%)	34 (12.9%) 5 (19.2%)	
Osteoporosis					0.261
No n, (%) Yes n, (%)	9 (3.6%) 0 (0%)	88 (34.8%) 13 (36.1%)	125 (49.4%) 15 (41.7%)	31 (12.3%) 8 (22.2%)	
Death					0.05
No n, (%) Yes n, (%)	9 (3.1%) 0 (0%)	101 (35.3%) 0 (0%)	139 (48.6%) 1 (33.3%)	37 (12.9%) 2 (66.7%)	

**Fig. 2:**
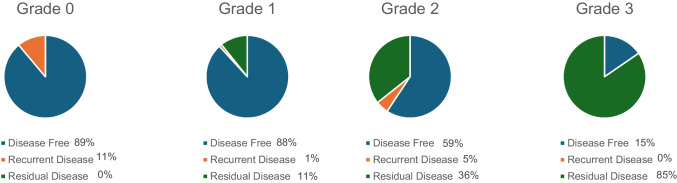
Pie charts representing the PAs outcome, according to PANOMEN-3 grades

**Fig. 3:**
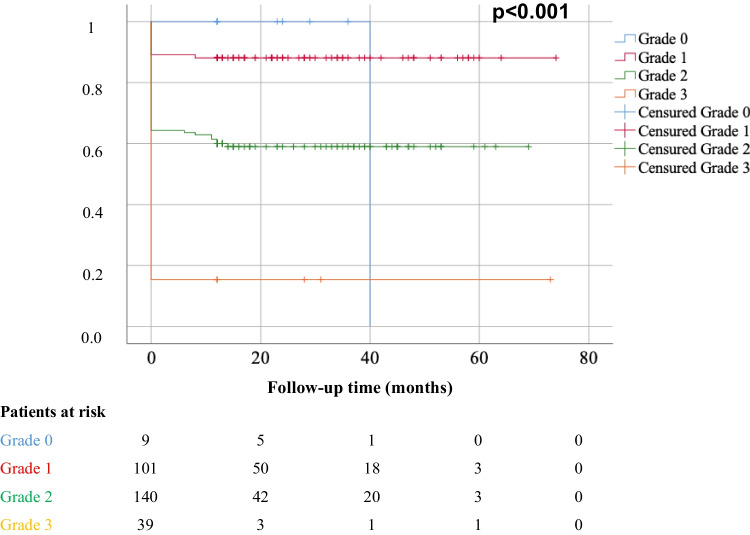
Cumulative disease-free survival time, Kaplan-Meir plot. Disease-free survival time in PANOMEN-3 grade 3 vs grade 0 *p* = 0.009; grade 3 vs grade 2 *p* < 0.001, grade 2 vs grade 1 *p* = 0.015

The PANOMEN-3 classification significantly correlated with pituitary adenoma outcome (*p* < 0.001), as shown in Fig. 2. Of the 9 patients classified as PANOMEN-3 grade 0, eight were disease-free (88.9%), none had a post-surgery residual, and disease recurrence occurred in a single patient (11.1%). Among the 101 patients classified as PANOMEN-3 grade 1, 89 were disease free (89.1%), 11 had post-surgery residual disease (10.9%) and 1 experienced disease recurrence (1%). Among the 140 patients classified as PANOMEN-3 grade 2, 83 were disease free (59.3%), 50 had post-surgery residual disease (35.7%) and 7 experienced disease recurrence (5%). Among the 39 patients classified as PANOMEN-3 grade 3, 6 were disease free (15.4%), 33 had a post-surgery residual disease (84.6%), and none experienced disease recurrence (0%). Grades 1, 2 and 3 of the PANOMEN-3 score were associated with shorter disease-free survival interval as compared to grade 0 PANOMEN-3 score, as shown in Fig. 3. Median disease-free survival was 23 months (IQR: 20, ranges: 12–40) for patients classified as grade 0; 19 months (IQR: 22, ranges: 0–74) in patients classified as grade 1; 12 months (IQR: 30, ranges: 0–69) for patients classified as grade 2 and 0 month (IQR: 0, ranges: 0–73) for patients classified as grade 3. One patient with PANOMEN-3 grade 2 died, while, in two patients with PANOMEN-3 grade 3 died.

We investigated separately each item of the PANOMEN-3 score to ascertain which are the most significantly associated to recurrence/residual disease, as reported in Table 2. We found a significant higher frequency of recurrence/residual disease in patients with biochemical active disease (*p* < 0.001) at the time of the application of the PANONEN-3 classification; in patients with giant pituitary adenoma (*p* = 0.002); in patients with visual field defect and/or cranial nerve palsy (*p* = 0.005); in patients carriers cavernous sinus invasive pituitary adenoma (*p* < 0.001); in patients carrying a post-operative residual disease (*p* < 0.001); and in patients with high-risk subtype or increased proliferative pituitary adenoma (p = 0.036).

**Table 2:** Analysis of items of the PANOMEN-3 classification in predicting the different outcomes (disease-free, residual disease, recurrent disease) at last follow-up. Univariate analysis. N.: number

	Disease-free	Recurrence and residual disease	*p*
Phenotype NPFA and Prolactinoma n, (%) GH secreting adenoma and TSH-secreting adenoma n, (%) ACTH-secreting n, (%)	114 (61%) 48 (70.6%) 24 (70.6%)	73 (39%) 20 (29.4%) 10 (29.4%)	0.264
Secretory status Normal or biochemically controlled n, (%) Not biochemically controlled n, (%)	175 (72.6%) 11 (22.9%)	66 (27.4%) 37 (77.1%)	< 0.001
Hypopituitarism Absent n, (%) Partial without adrenal or AVP deficiency n, (%) Hypopituitarism with adrenal or AVP deficiency n, (%)	109 (66.9%) 19 (79.2%) 58 (56.9%)	54 (33.1%) 5 (20.8%) 44 (43.1%)	0.073
Tumor size Microadenoma n, (%) Macroadenoma n, (%) Giant adenoma n, (%)	41 (82%) 141 (62.1%) 4 (33.3%)	9 (18%) 86 (37.9%) 8 (66.7%)	0.002
Mass effect Absence n, (%) Visual field defect and/or cranial nerve palsy n, (%) Cerebrospinal fluid (CSF) leak n, (%)	131 (70.1%) 55 (53.9%) 0 (0%)	56 (29.9%) 47 (46.1%) 0 (0%)	0.005
Invasion Not-invasive tumors n, (%) Tumor with invasion of the cavernous sinus n, (%)	158 (74.9%) 28 (35.9%)	53 (25.1%) 50 (64.1%)	< 0.001
Residual tumor Absent n, (%) Present n, (%)	185 (94.9%) 1 (1.1%)	10 (5.1%) 93 (98.9%)	< 0.001
Histopathology Not high risk and not proliferative adenoma n, (%) High-risk subtypes n, (%) Increased proliferation subtypes n, (%)	176 (66.4%) 10 (43.5%) 0 (0%)	89 (33.6%) 13 (56.5%) 1 (100%)	0.036
Genetic syndrome Absent n, (%) Present n, (%)	185 (64.5%) 1 (50%)	102 (35.5%) 1 (50%)	0.587

A part of PANOMEN-3 classification, the recurrence or persistence of post-surgery residual of PAs were significantly more frequent in cases with Mib-1 > 7% (*p* = 0.019), in cases with > 2 mitosis/HPFs (*p* = 0.006), and in cases underwent multiple surgical resections (*p* < 0.001), as showed in Table 3.

**Table 3:** Factors associated with different outcomes (disease-free, residual disease, recurrent disease). Univariate analysis. N.: number, *: data available for 286 patients

	Disease-free	Recurrence and residual disease	*p*
Gender Male n, (%) Female n, (%)	91 (61.5%) 95 (67.4%)	57 (38.5%) 46 (32.6%)	0.296
Age median (IQR)	53 (17)	54 (27)	0.155
Histologic adenoma subtypes Gonadotroph n (%) Corticotroph, n (%) Densely granulated somatotroph n, (%) Sparsely granulated somatotroph n, (%) Mammosomatotroph n, (%) Lactotroph n, (%) Thyrotroph n, (%) Mature plurihormonal PIT-1 lineage n, (%) Immature PIT-1 lineage n, (%) Crooke cell n, (%)	77 (62.6%) 36 (67.9%) 33 (68.8%) 5 (50%) 2 (100%) 17 (50%) 7 (100%) 8 (80%) 1 (100%) 0 (0%)	46 (37.4%) 17 (32.1%) 15 (31.3%) 5 (50%) 0 (0%) 17 (50%) 0 (0%) 2 (20%) 0 (0%) 1 (100%)	0.153
Clinical adenoma subtypes Non secreting n, (%) GH-secreting n, (%) TSH-secreting n, (%) ACTH-secreting n, (%) PRL-secreting n, (%)	97 (63.4%) 45 (69.2%) 3 (100%) 24 (70.6%) 17 (50%)	56 (36.6%) 20 (30.8%) 0 (0%) 10 (29.4%) 17 (50%)	0.197
Mib-1 * < 3% n, (%) 3% > Mib-1 ≤ 7% Mib-1 > 7%	174 (66.9%) 11 (45.8%) 0 (0%)	86 (33.1%) 13 (54.2%) 2 (100%)	0.019
p53 expression Negative n, (%) Positive n, (%)	169 (66.5%) 18 (51.4%)	85 (33.5%) 17 (48.6%)	0.08
Mitosis * < 2/HPFs n, (%) > 2/HPFs n, (%)	183 (66.1%) 2 (22.2%)	94 (33.9%) 7 (77.8%)	0.006
Adenoma growth pattern Not invasive n, (%) Invasive n, (%)	158 (74.9%) 28 (35.9%)	53 (25.1%) 50 (64.1%)	< 0.001
PANOMEN-3 Score Grade 0 n, (%) Grade 1 n, (%) Grade 2 n, (%) Grade 3 n, (%)	8 (88.9%) 89 (88.1%) 83 (59.3%) 6 (15.4%)	1 (11.1%) 12 (11.9%) 57 (40.7%) 33 (84.6%)	< 0.001
Numbers of surgical resections Single n, (%) Two or more n, (%)	176 (68.8%) 10 (30.3%)	80 (31.2%) 23 (69.7%)	< 0.001
Pre-surgical medical therapy No n, (%) Yes n, (%)	157 (65.1%) 29 (60.4%)	84 (34.9%) 19 (39.4%)	0.532
Deaths No n, (%) Yes n, (%)	186 (65%) 0 (0%)	100 (35%) 3 (100%)	0.019

We investigated the correlation between PANOMEN-3 grades and treatments at last follow-up, according to the different PA clinical subtypes, as depicted in Table 3. No significant differences were identified among the therapeutic interventions and PANOMEN-3 grades in patients with non-secreting PAs, TSH-secreting PAs, and ACTH-secreting PAs. In the cohort of 65 patients diagnosed with acromegaly, patients cured only through surgery were more frequently classified as grade 1 (19: 54.3%) and grade 2 (14: 40%) of the PANOMEN-3 score; patients who needed first line medical therapies (DAs and fg-SRLs) after neurosurgery were more frequently classified as grade 2 of the PANOMEN-3 score (DAs: 51.7%, fg-SRLs: 64.3%); and patients who underwent neurosurgery and needed second line therapies (second generation SRLs or GHR antagonist) were more frequently classified as grade 2 (66.3%) and as grade 3 (33.3%). Among the 34 patients with prolactin secreting Pas operated, patients who were cured through surgery 6 were classified as grade 0 of the PANOMEN-3 score (31.6%), 10 as grade 1 of the PANOMEN-3 score (52.6%), and 3 as grade 2 (15.8%). One operated patient controlled with dopamine agonists was classified as grade 1 (8.3%), 7 as grade 2 (50%), and 6 as grade 3 (42.9%). A single patient with a grade 2 of the PANOMEN-3 score underwent combination therapy (DA plus fg-SRLs) Table 4.

**Table 4:** Treatment at last follow-up according to PANOMEN-3 grades. Univariate analysis

Adenomas types	Grade 0	Grade 1	Grade 2	Grade 3	p
Non-secreting Observation, n (%) DA n, (%) fg-SRLs, n (%)	2 (1.3%) 0 (0%) 0 (0%)	61 (40.7%) 1 (50%) 0 (0%)	71 (47.3%) 1 (50%) 0 (0%)	16 (10.7%) 0 (0%) 1 (100%)	0.215
GH-secreting Observation, n (%) DA n, (%) fg-SRLs n, (%) Pasireotide Lar or Pegvisomant or combinations, n (%)	0 (0%) 0 (0%) 0 (0%) 0 (0%)	19 (54.3%) 1 (14.3%) 2 (14.3%) 0 (0%)	14 (40%) 5 (71.4%) 9 (64.3%) 6 (66.6%)	2 (5.7%) 1 (14.3%) 3 (21.4%) 3 (33.3%)	0.018
TSH-secreting Observation, n (%)	0 (0%)	3 (100%)	0 (0%)	0 (0%)	n.a
ACTH-secreting Observation, n (%) Steroidogenesis inhibitors, n (%)	0 (0%) 0 (0%)	4 (13.3%) 0 (0%)	21 (70%) 2 (50%)	5 (16.7%) 2 (50%)	0.266
PRL-secreting Observation, n (%) DA n, (%) DA + fg-SRLS n, (%)	6 (31.6%) 1 (7.1%) 0 (0%)	10 (52.6%) 0 (0%) 0 (0%)	3 (15.8%) 7 (50%) 1 (100%)	0 (0%) 6 (42.9%) 0 (0%)	0.001

### Logistic regression

The final model to predict PAs recurrence/persistence of post-operative residual at last follow-up was obtained from the stepwise selection process Table 5. The grades 1, 2 and 3 of the PANOMEN-3 score independently increased the risk of recurrent or residual disease with an odd respectively of 1.4 (95%IC: 1.2–1.7), 1.5 (95%IC: 1.2–1.9) and of 5.7 (95%IC: 2.7–12.5). Among the items of PANOMEN-3 score, the not-biochemically controlled secretory status and the tumor invasiveness were identified as independently risk factors (respectively OR: 2, 95%IC: 1.5–2.8 p = 0.028 and OR:8.9 95%IC:4.2–18.5 p = 0.046) for the outcome at last follow-up (residual/recurrence of pituitary adenomas). The absence of post-operative residual disease was identified as a protective factor (OR: 0.05, 95%IC: 0.028–0.095, p < 0.001) for the outcome at last follow-up (residual/recurrence of pituitary adenomas).

**Table 5:** Logistic regression model. Abbreviations: OR: odds ratio, IC: Confidence interval

	*p*	OR	95%CI	Beta
Secretory status Not-biochemically controlled	0.046	8.9	4.2–18.5	−2.5
Tumor size Microadenoma Macroadenoma Giant adenoma	0.758 0.721 0.961	2.8 3.2	0.9–5.9 0.9–11.2	0.964 0.12
Mass effect Absence n Visual field defect and/or cranial nerve palsy Cerebrospinal fluid (CSF) leak	Ref 0.872 -	1.3 -	0.89–3.3 -	−0.409 -
Invasive growth pattern	0.028	2	1.5–2.8	0.739
Absence of residual tumor	< 0.001	0.05	0.028–0.095	−7.885
Histopathology Not high risk and not proliferative adenoma High-risk subtypes Increased proliferation subtypes	0.848 0.999 0.999	1.58 1	0.95–2.45 0.98–1.1	0.001 −0.262
PANOMEN-3 Score Grade 0 Grade 1 Grade 2 Grade 3	Ref 0.003 < 0.001 < 0.001	1.4 1.5 5.7	1.2–1.7 1.2–1.9 2.7–12.5	−3.4 −3.5 −2
Mib-1 < 3% 3% > Mib-1 ≤ 7% Mib-1 > 7%	Ref 0.999 0.999	1.4 1	0.9–2.2 0.9–1	−18.9 −18.8
Mitosis	0.719	2.9	0.87–10.1	- 0.927
Multiple surgical resections	< 0.001	5	2.3–11.1	1.63

Furthermore, multiple surgery pituitary adenoma resections have been associated with an increased risk of recurrent or residual disease (respectively; and OR: 5 95%IC: 2.3–11.1, p < 0.001).

## Discussion

In this study we investigated the efficacy of the PANOMEN 3 classification in predicting the outcomes of PAs and in stratifying the risk of disease recurrence and progression. The results of this study demonstrated a correlation between the higher grades of the PANOMEN-3 classification and an increased risk of recurrent/residual disease, as well as a reduced disease-free survival.

Over the years, several prognostic classifications of PAs have been attempted by scientific associations and societies. In 2004, the WHO distinguished typical and atypical adenomas, and pituitary carcinoma, thus acknowledging the variable clinical presentations and biological behaviors associated with these neoplasms [6]. Atypical adenomas were diagnosed with high mitotic activity, excessive p53 immunoreactivity, proliferative index (Ki-67) above 3%, and invasive growth [6].

In 2017, the WHO released a revised classification of Tumours of Endocrine Organs [21], in which the definition of atypical adenoma was removed, as studies failed to find significant difference in recurrence risk and rate between typical and atypical adenomas [22–24]. Furthermore, the 2017 WHO Classification of the Tumours of Endocrine Organs introduced the analysis of transcription factors and differentiated pituitary adenoma in those with low probability and those with high probability of recurrence, and malignant (metastatic) tumor, and suggested to integrate the nomenclature “pituitary neuroendocrine tumors” to better define the heterogeneous behavior of these neoplasms [21]. Pituitary adenomas with elevated proliferative activity, sparsely granulated somatotroph adenomas, lactotroph adenomas in men, silent corticotroph adenomas, Crooke cell adenomas and plurihormonal PIT-1 positive adenomas were identified as those with probability of recurrence [21].

The 2022 WHO Classification of the Tumours of Endocrine Organs revised the class of risk for pituitary adenomas/neuroendocrine tumors, according to the expression of transcription factors, suggesting a more aggressive clinical behavior in sparsely granulated somatotroph tumor, immature Pit-1 lineage tumor and acidophil stem cell tumors (among Pit-1 lineage derived tumor); sparsely granulated corticotroph tumor and Crooke cell tumor (among T-PIT lineage derived tumor); and null cell adenoma (among tumor without distinct cell lineage) [8] However, the WHO classification did not provide PA grading and a staging. Thus, few studies were conducted to design a prognostic grading and staging score, that would predict the outcome of PAs and the risk of residual disease or recurrence.

In 2013, a five-tiered classification for pituitary adenomas was proposed, with tiers designated according to invasion and proliferative characteristics [5]. This clinicopathological classification has been validated in several real-life studies, showing that higher grades of this classification (grade 1b and 2b) predict progression-free survival and the risk of recurrence and progression [25–29].

More recently, the PANOMEN-3 classification integrated clinical, biochemical, morphological, pathological and genomic features into a single prognostic score that could predict the severity of PAs. To date, the PANOMEN-3 classification has been validated in two retrospective cohort study, providing that increased PANOMEN-3 grades correlated with the recurrence/progression rates: both studies proved that grade 3 of the PANOMEN-3 classification was associated with a high risk of recurrence and shorter disease-free survival [10–30].

In this real-life and single center study, we examined the association between the PANOMEN-3 classification and outcomes of 289 consecutive patients. To our knowledge, we now show for the first time that the PANOMEN-3 classification may predict the outcome of patients with PAs. Grades 1, 2 and 3 score correlated with a progressive increased risk of residual disease or recurrence, as well as a reduced disease-free survival period. Grade 1 was associated with a 1.4-fold elevated risk of residual/recurrence disease, the grade 2 was associated with a 1.5-fold high risk of residual/recurrence disease, and the grade 3 was associated with a 5.7 times higher risk of residual/recurrence disease. Therefore, our results support the application of the PANOMEN-3 score in real-life clinical practice. This study investigated also the correlation between the PANOMEN grades and the occurrence of systemic comorbidities. As higher grades of PANOMEN-3 classification resulted associated to poor controlled disease, we would like to investigate the potential correlation with an increased frequency of pituitary adenoma related comorbidities. However, in our cohort, the occurrence of diabetes mellitus, arterial hypertension, major cardiovascular events, second neoplasms, and osteoporosis was not associated to the grades of the PANOMEN-3 classification. Interestingly, for the first time to our knowledge, our finding provided that the grade 3 of the PANOMEN-3 score were associated to an increased mortality, suggesting that the grade 3 of the PANOMEN-3 score well represents a subgroup of patients with aggressive pituitary adenomas.

Furthermore, our findings indicated that elevated PANOMEN-3 scores were associated with the need of post-surgery medical treatments to manage somatotroph and lactotroph tumors. Patients with acromegaly and classified as grade 1 were in emission in most patients undergoing surgery, patients classified as grade 2 achieved disease control mainly with first-line medical therapies (DA and fg-SRLs), and patients classified as grade 3 required first or second-line medical therapies (Pasireotide Lar or GHR-antagonist). Patients with lactotroph tumors classified as grades 0 and 1 were in remission in most cases with surgery, patients classified as grades 2 and 3 achieved disease control mainly with post-surgery treatment with DAs, and in a single case with combination DA plus fg-SRLs.

In our study, we investigated separately the items included in the PANOMEN-3 scores to ascertain which were the most significant. As expected, we found that not-biochemically controlled patients were at significant high risk for persistence of residual/recurrent disease at last follow-up. This finding may be supported by the presence of post-surgery residual disease. In fact, on the other hand, the absence of post-surgery residual was identified as a protective factor, for the occurrence of pituitary adenoma recurrence or persistence of residual disease. These data were in accordance with those reported also in the multicenter Spain study that provided that the presence of residual tumor, the persistence of active secretory status and the presence of hereditary syndrome [10].

Moreover, this study shows that the risk of residual/recurrent disease is higher in patients who underwent multiple surgical resections, in accordance with an invasive tumor growth. According to our results, in fact invasive tumor growth rather than proliferation is recognized as a risk factor for adenoma recurrence or persistence of residual disease. In fact, expert surgeons (as defined by criteria of the pituitary tumor centers of excellence (PTCOE) [31] there is a good chance of removal of encapsulated and non-invasive PAs even if proliferative. In fact, in a surgical cohort of 306 patients with PAs, we reported a lower rate of surgical radical resection in invasive non-proliferative PAs than in non-invasive but proliferative PAs [28]. In this study, total surgical resection was achieved in 90.4% of non-invasive and non-proliferative PAs, in 92.4% of non-invasive but proliferative PAs, in 46.4% of invasive but non-proliferative PAs and in 43% of invasive and proliferative PAs [28].

The main limitation of our study was the retrospective design, that should be considered for the interpretation of the results, and that may be impacted on the enrollment of patients in this study. Another limitation of this study is the inclusion in the study cohort only of patients underwent neurosurgical resection for the removal of pituitary adenoma at the Neurosurgery Division at Gemelli University hospital. In this view, this may represent a selection bias, according to the high frequency of macroadenoma with respect to microadenoma and secreting pituitary adenomas with respect of not-secreting ones. However, this potential selection bias did not impact on the primary objective of the study that was to assess the utility of the PANOMEN-3 classification in predicting the outcome of pituitary adenomas, as this study had not an epidemiology design. Another limitation of this study is due to the short-term follow-up that reflects our aim to apply the 2022 WHO classification of Tumours of Endocrine Organs and the PANOMEN-3 score in prognostic stratification of PAs. Another limitation of this study is the absence of genetic analysis for the whole cohort of patients. In fact, genetic testing was performed as recommended [32] in patients with a family history of pituitary tumours or relevant syndromic disease, in patients with early‐onset pituitary tumours, or in patients with clinical features suggestive for a syndromic setting.

Despite these limitations, this study reports a large single center cohort of 289 patients evaluated and treated by a unique team of physicians devoted to pituitary disease, following standardized procedures for diagnosis, treatment and follow-up. Therefore, our results appear to validate the PANOMEN-3 score in a real-life large cohort study, finding a univocal predictive role of PANOMEN-3 score in recurrence risk- and disease-free survival.

This study demonstrates that the higher grades of the PANOMEN-3 score predicted a progressively increasing risk for post-surgery residual and recurrence of pituitary adenomas, suggesting the efficacy of the PANOMEN-3 score in real-life clinical practice. This newly proposed comprehensive classification integrating biochemical, pathologic, imaging and clinical information is shown here to inform a score facilitating optimal patient follow-up and effective post-surgical management of residual disease and treatment strategies.

## Data Availability

No datasets were generated or analysed during the current study.
